# The 4Ds of ectopic ACTH syndrome: diagnostic dilemmas of a difficult disease

**DOI:** 10.20945/2359-3997000000129

**Published:** 2019-04-15

**Authors:** Marcelo Vieira-Corrêa, Débora Moroto, Giovanna Carpentieri, Igor Veras, Claudio E. Kater

**Affiliations:** 1 Universidade Federal de São Paulo Departamento de Medicina Escola Paulista de Medicina Universidade Federal de São Paulo São Paulo SP Brasil Unidade de Adrenal e Hipertensão, Disciplina de Endocrinologia e Metabologia, Departamento de Medicina, Escola Paulista de Medicina, Universidade Federal de São Paulo (EPM/Unifesp), São Paulo, SP, Brasil

## Abstract

Cushing’s syndrome (CS) is an uncommon condition that leads to high morbidity and mortality. The majority of endogenous CS is caused by excessive ACTH secretion, mainly due to a pituitary tumor – the so-called Cushing’s disease (CD) – followed by ectopic ACTH syndrome (EAS), an extra-pituitary tumor that produces ACTH; adrenal causes of CS are even rarer. Several methods are used to differentiate the two main etiologies: specific laboratory tests and imaging procedures, and bilateral inferior petrosal sinus sampling (BIPSS) for ACTH determination; however, identification of the source of ACTH overproduction is often a challenge. We report the case of a 28-year-old woman with clinical and laboratory findings consistent with ACTH-dependent CS. All tests were mostly definite, but several confounding factors provoked an extended delay in identifying the origin of ACTH secretion, prompting a worsening of her clinical condition, with difficulty controlling hyperglycemia, hypokalemia, and hypertension. During this period, clinical treatment was decisive, and measurement of morning salivary cortisol was a differential for monitoring cortisol levels. This report shows that clinical reasoning, experience and use of recent methods of nuclear medicine were decisive for the elucidation of the case.

## INTRODUCTION

Cushing’s syndrome (CS) is a cluster of clinical signs and symptoms secondary to cortisol excess. It may occur due to chronic glucocorticoid administration or, less frequently, due to endogenous production ( 1 ). Endogenous CS is classified according to ACTH dependence: ACTH-independent CS (primary adrenal disease) corresponds to less than 20% of cases, whereas the ACTH-dependent form (secondary disease) is much more prevalent (~80%). Confirmation of cortisol autonomy, identification of its etiology, and proper treatment remain challenges ( 1 ).

The major cause of CS, encompassing 60-70% of all cases, is an ACTH-producing pituitary tumor (Cushing’s disease [CD]), generally a microadenoma. Ectopic ACTH syndrome (EAS), an ACTH-producing extra-pituitary tumor of several origins, is the second endogenous cause, responsible for 5-10% of cases ( 1 , 2 ). EAS may also result from an ectopic CRH-producing tumor ( 1 ). The most frequent neoplasms associated with EAS are small-cell lung carcinoma and other neuroendocrine tumors of the lungs, thymus and pancreas ( 3 ). Occasionally, a thyroid medullary carcinoma, pheochromocytoma, gastrinoma, or prostate carcinoma may produce the EAS ( 3 ).

Classical stigmata of CS are present in both CD and EAS. However, metabolic derangements and several other unspecific features are more prevalent in EAS, such as a rapid disease progression ( 4 ), intense proximal muscle weakness, severe hypertension and hypokalemia ( 5 ), and marked hyperpigmentation secondary to higher plasma ACTH levels (usually >100 pg/mL) ( 6 ).

A major challenge in the EAS is to determine the origin of ACTH excess. Up to 50% of these tumors may escape detection by chest and abdominal computerized tomography (CT) and/or magnetic resonance imaging (MRI) ( 3 , 7 ), and remain “occult”. Simultaneous or coupled use of plain (CT/MRI) and functional imaging (scintigraphy) are useful for these undetermined cases.

## CASE REPORT

A 28-year-old Caucasian woman was referred to the Adrenal Outpatient Clinic at EPM/UNIFESP because of progressive and rapid darkening of the skin and recent weight gain. In several weeks, her face turned plethoric, oily and hairy with acne. Within the past 6 months, the patient noticed a further progression of weight gain especially in her belly, a “buffalo hump”, several purple striae in her thighs and abdomen, and intense proximal muscle weakness.

The patient’s past medical history was unremarkable except for the presence of endometriosis, which has been treated for the past two years with the continuous use of a combined oral contraceptive (ethinylestradiol 30 mcg plus gestodene 75 mcg), making her amenorrheic ever since. She denied other comorbidities and the use of any other medications (including corticosteroids) and recreational drugs.

Initial physical exam disclosed weight of 66.2 kg and height of 167 cm (BMI = 23.7 kg/m^2^). The patient has a full-blown cushingoid appearance with moderate central obesity, discrete cervicodorsal adiposity, plethoric “moon facies”, diffuse scalp balding, increased hair in androgenic areas, facial and dorsal acne, skin thinning with squamation, purple striae in the abdomen and root of the thighs (some wider than 1 cm), and muscle hypotrophy in the legs, with a grade III diminished strength.

### Investigation and treatment

Initial laboratory results ( Table 1 ) revealed hypercortisolism with increased ACTH levels, hyperandrogenism and an otherwise normal metabolic profile. These results typically supported the syndromic diagnosis of ACTH-dependent CS.

**Table 1:** Results of initial hormonal evaluation and reference values

**Hormonal test**	**Result**	**Reference values**
Testosterone	98	< 10 – 75 ng/dL
Dehydroepiandrosterone sulfate (DHEA-S)	504	96 – 512 mcg/dL
Dehydroepiandrosterone (DHEA)	1,200	100 – 1,200 ng/dL
Androstenedione	1,140	40 – 410 ng/dL
Follicle-stimulating hormone (FSH)	0.16	3.5 – 12.5 mUI/mL
Luteinizing hormone (LH)	0.07	2.4 – 12.6 mUI/mL
Corticotropin (ACTH)	138	7.2 – 63 pg/mL
Basal Serum cortisol	33.1	6.2 –19.4 mcg/dL
23h-Salivary cortisol	6,040	< 250 ng/dL
Post-1mg DST Salivary cortisol	1,110	< 50 ng/dL
Post-1mg DST Serum cortisol	13	< 2.5 mcg/dL

**Figure 1:**
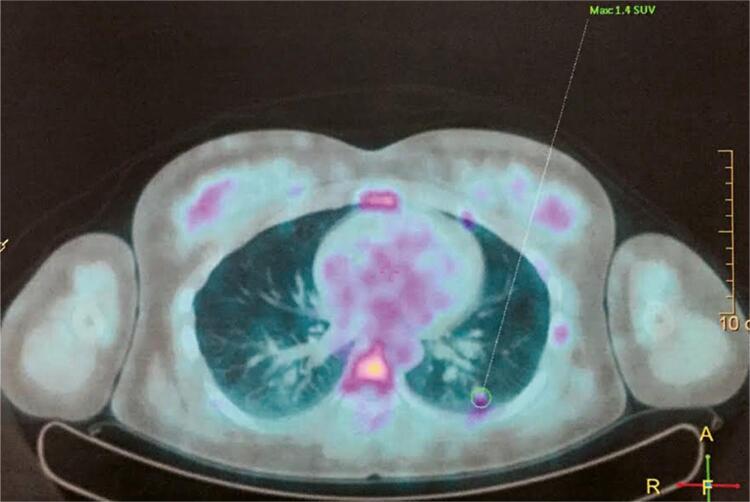
A suspicious lung nodule investigated with 18F-FDG PET-CT showing discrete glucose uptake (SUV of 1.4).

To investigate for the possibility of CD, a pituitary MRI proved non-informative. Subsequently, a chest CT and an MRI showed a 0.8 cm nodular lesion in the inferior lobe of the left lung. The suspicious nodule was then investigated with a Fluor-deoxyglucose scintigraphy (^18^F-FDG PET-CT; Figure 1 ), showing a discrete glucose uptake (SUV of 1.4). Additional investigation with an ^111^In-Pentetreotide scintigraphy (Octreoscan^®^) was negative.

After consulting with the thoracic surgeons and radiologists in the adrenal unit, it was concluded that the lesion characteristics were possibly of vascular origin and unrelated to the disease. Needle biopsy and surgical removal were both inadvisable.

Because a previous thyroid ultrasonography was positive for the presence of two solid nodules of 1.3 and 1.2 cm in diameter, attention was directed toward the possibility of a medullary thyroid carcinoma being the origin of ACTH excess. Fine-needle aspiration biopsy was performed, and the nodules were characterized as Bethesda II. ACTH and calcitonin levels in the fluid wash were negative.

A second pituitary MRI employing a 3-Tesla machine and dynamic study revealed a 6mm cystic lesion in the upper posterior right portion of the gland. Although considered of low suspicion potential, it prompted a detailed reinvestigation of the pituitary as the origin of the disease.

We have not employed the high-dose 8 mg DST, and have virtually abandoned it because it generates up to 30% false results and a high rate of discordance with the CRH stimulation test ( 1 ).

Therefore, the patient underwent a bilateral inferior petrosal sinus sampling (BIPSS) for ACTH measurements ( Table 2 ). Prolactin levels were used to confirm adequate catheterization, and the ACTH/prolactin ratio was used to interpret the results. Although the results were somewhat abnormal (RPS/PV ratio of 3.87 at 3 min), the final interpretation considered the pituitary lesion incidental and dismissed it as the source of the ACTH excess.

**Table 2:** Bilateral inferior petrosal sinus sampling (BIPSS)

**Time**	**ACTH (pg/ml) | PRL (ng/mL)**			**LPS/PV**	**RPS/PV**

	**Left petrosal sinus (LPS)**	**Right petrosal sinus (RPS)**	**Peripheral vein (PV)**		
Basal (0 min)	170 | 16.7	293 | 22.8	365 | 24.5	0.46	0.8
1 min	413 | 23.3	393 | 19.5	157 | 21.6	2.61	2.48
3 min	317 | 19.0	457 | 18.4	117 | 19.5	1.78	3.87
5 min	465 | 18.9	420 | 16.6	352 | 18.9	1.32	1.19
10 min	458 | 17.5	513 | 15.1	441 | 16.5	1.03	1.16

Eight months after the initial symptoms, the patient experienced spontaneous and significant clinical remission that lasted for 3 months. During this period, serum, salivary and urinary cortisol reduced to near-normal levels. Afterward, however, symptomatology resumed very dramatically, associated with increased blood pressure, severe and symptomatic hypokalemia and hyperglycemia. The patient was admitted to the endocrine ward to receive regular IV insulin and potassium chloride in high doses, associated with oral spironolactone. While in the hospital, the patient had a spontaneous L2-vertebral fracture, developed severe and disabling muscle weakness, episodic psychosis, and primary hypothyroidism. The relapsed clinical picture was so intense and refractory to conventional therapy that a bilateral adrenalectomy was considered as an emergency therapy.

**Figure 2:**
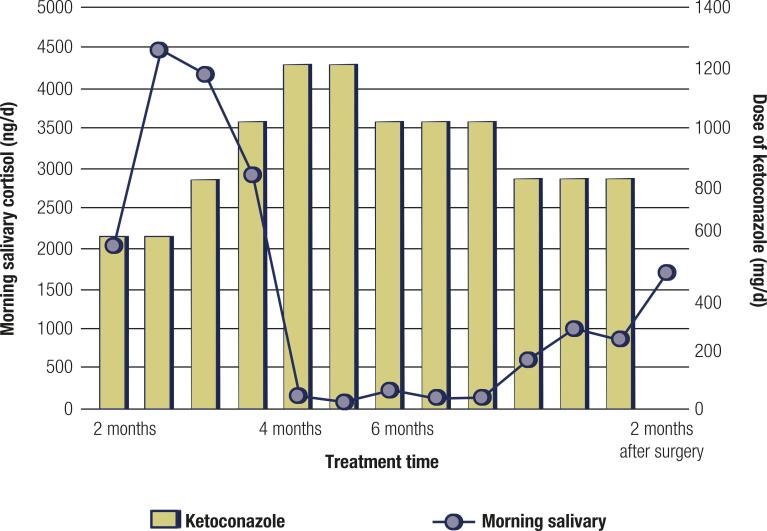
Morning salivary cortisol levels x Ketoconazole (KCZ) dose (mg/day).

However, insulin pump infusion, high-dose spironolactone (200-300 mg/day), zoledronic acid, and nandrolone decanoate resulted in a progressive improvement of the clinical picture within the next several weeks, in particular of muscle weakness. Nevertheless, we decided to perform a “pharmacologic adrenalectomy” via steroid-enzyme inhibition with ketoconazole (KCZ). After an initial oral dose of 200mg/day for a few days, the treatment schedule scaled up to 1.2 g/day and plateaued at 1 g/day. During this treatment period, morning salivary cortisol levels were measured at weekly intervals to ascertain the effectiveness of cortisol blockade by KCZ ( Figure 2 ).

During the next 4 months, serum and salivary cortisol levels decreased substantially on an average daily dose of 600 mg/day of KCZ and clinical manifestations improved. On continuous KCZ therapy, clinical effectiveness was confirmed when the patient began demonstrating signs of adrenal insufficiency, requiring introduction of oral prednisolone (2.5-5.0 mg/day).

**Figure 3:**
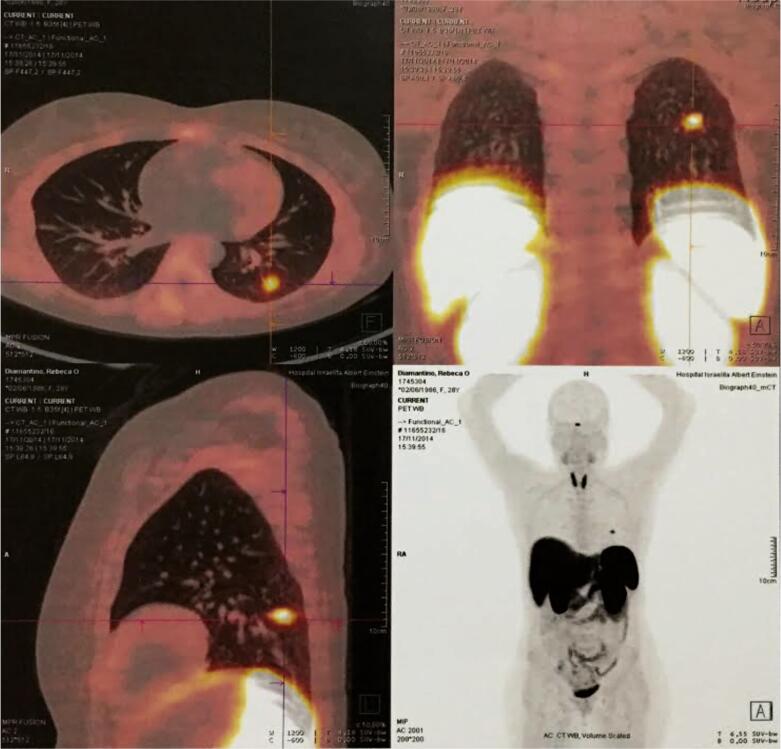
68Ga-DOTA-TATE PET-CT showing anomalous tracer uptake: a regular, solid, non-calcified lung nodule, located between the superior and basal posterior segments of the left lung inferior lobe that measured 1.3 x 0.9 cm in diameter, with an SUV of 6.1, consistent with a tumor of neuroendocrine origin (carcinoid tumor).

Following pharmacologic control of the disease, etiological investigation continued. A somatostatin receptor imaging employing ^68^Gallium PET-CT (^68^Ga-DOTA-TATE) was obtained and disclosed an anomalous tracer uptake in the same region previously seen on ^18^F-FDG-PET-CT: a regular, solid, non-calcified lung nodule, located between the superior and basal posterior segments of the left inferior lobe of the right lung, that measured 1.3 x 0.9 cm in diameter, with an SUV of 6.1 ( Figure 3 ). This finding was consistent with a tumor of neuroendocrine origin (a carcinoid tumor), reinforcing the diagnosis of an ectopic ACTH-producing lung tumor.

The patient underwent a left inferior lobe lung resection 22 months after the beginning of her clinical symptoms. A prompt drop in plasma ACTH and serum cortisol concentrations followed within 1 hour after tumor resection, reaching undetectable cortisol levels 24h later ( Table 3 ).

**Table 3:** Pre- and post-operative levels of ACTH and cortisol

**Hormonal test**	**ACTH**	**Cortisol**
Basal (Pre-Op)	102	19.2
Post-operative period		
1h	7.2	11.8
2h	1.5	8.8
4h	< 1.0	3.9
24h	< 1.0	0.57
2 months	–	6.6
3 months	–	3.6
4 months	26.9	3.4

Normal ranges: ACTH: 7.2 – 63 pg/mL; cortisol: 6.2 – 19.4 mcg/dL.

## OUTCOME AND FOLLOW-UP

Pathology examination revealed a well-differentiated neuroendocrine lung neoplasia: immunohistochemistry was positive for ACTH, chromogranin A, and synaptofisin; Ki-67 was positive in less than 5% of the cells.

Less than 3 months after surgery, the patient was free of symptoms and from glucocorticoid replacement therapy. Cortisol levels were within the normal range and responsive to suppression (1 mg DST < 1.0 mg/dL). The patient remains completely asymptomatic and hormonally controlled 2 years after surgery.

## DISCUSSION

The etiology of Cushing’s syndrome may occasionally be difficult to ascertain. In patients with EAS, identification of the source of autonomous production of ACTH may remain unknown (occult or indeterminate) in up to 19% of cases ( 2 ). The delay in establishing the origin of the disease and in controlling hypercortisolism may predictably result in severe consequences.

One potential problem in diagnosing CS is the occurrence of a cyclical pattern of cortisol excess. Although cyclic CS is observed more often in primary pigmented nodular adrenal hyperplasia/dysplasia (PPNAD), as well as in isolated micronodular adrenocortical disease, it may occur in any form of endogenous CS ( 1 ). The wax-and-wane pattern of symptoms raised the suspicion of cyclic CS in our patient.

Although the pre-test diagnostic probability of CD is higher than 80%, our team contemplated the less common EAS as the main diagnostic possibility for this patient from the beginning. The reasons contended were the sudden and rapid evolution of marked muscle weakness, and important hypokalemia and hyperpigmentation associated with significantly elevated plasma ACTH levels ( 2 ).

A pituitary MRI is usually the first imaging procedure indicated for the investigation of CS because of the major prevalence of CD. However, its results could be negative in identifying a pituitary microadenoma in up to 40% of patients. Subsequent chest and abdominal MRIs were suggestive of the presence of a suspicious left pulmonary nodule. A subsequent ^18^F-FDG PET-CT scan confirmed a moderate uptake by this very lung lesion ( 3 ).

In view of the modest ^18^F-FDG PET-CT outcome, an Octreoscan^®^ scintigraphy was performed, with a negative result. However, this procedure has a variable sensitivity for small lesions (between 25 and 80%, with a detection limit of 5 mm) ( 7 ). Moreover, hypercortisolism per se may suppress subtype-2 somatostatin receptor concentration and density, yielding false-negative results ( 1 ).

Once an ectopic source of ACTH excess was temporarily excluded, a new and more precise pituitary MRI, performed on recent generation 3-Tesla equipment, disclosed a previously unidentified 6 mm cystic lesion during the dynamic study. Although the pituitary lesion was unlikely to be responsible for the condition, a BIPSS for ACTH was performed, since it is considered the gold standard by which to establish the pituitary as the source of ACTH excess in CD, with 95% sensitivity and specificity ( 1 ).

Nevertheless, the results were not consistent: out of five sampling time-points, only a single one (at 3 min following desmopressin stimulation) disclosed a moderate ACTH and ACTH:prolactin ratio gradient; furthermore, overall ACTH levels were not significantly reduced. This result could reflect the presence of a cyclic disease and/or a mild hypercortisolism with no significant suppression of the remaining non-tumorous corticotrophs at that given moment. Yet another even rarer possibility was contemplated: co-production of CRH by the ectopic tumor stimulating normal ACTH-producing pituitary cells, leading to a false-positive BIPSS result ( 8 ).

After a prolonged and distressing period during which no definitive etiological diagnosis was achieved, we were able to employ a recently available scintigraphy procedure to localize tumors of neuroendocrine origin (with excellent accuracy to identify ectopic ACTH-producing tumors [ 7 ]): ^68^Ga-DOTA-TATE PET-CT (5). The result was an image with a significant tracer uptake (SUV of 6.1) located in the same lung area previously identified on the chest CT/MRIs and FDG PET-CT. Given the increased activity of the lesion and the higher accuracy of DOTA-TATE (82% sensitivity and near-100% specificity, even for lesions smaller than 10 mm [9]), this lesion was definitively considered the source of ACTH excess in our patient.

The histological findings were consistent with a neuroendocrine tumor, one of the more common etiologies of EAS ( 10 ). Our patient had classic features of a bronchial carcinoid neoplasia: young age, nonsmoker, a well-differentiated low-grade tumor, and usual immunohistochemical markers for ACTH, chromogranin A and synaptofisin. The patient underwent surgical tumor resection, the conventional treatment for bronchial carcinoid. The 5-year survival rate for typical carcinoid ranges from 92 to 100% ( 11 ).

Pharmacologic treatment of ectopic ACTH-producing neoplasms is usually transitory, to achieve normocortisolemia and improve clinical status until the occult tumor is found and surgically removed. Enzyme inhibitors of the steroid cascade are very effective for this purpose, although treatment with cabergoline, octreotide, and other ACTH suppressors may be effective as well ( 12 , 13 ). Any of these drugs has a chance for side effects, and availability may be limited.

Pharmacologic intervention with ketoconazole (KCZ) was pivotal to control the recurring severe clinical manifestations by blocking excessive cortisol production. This decision was made at a point when etiologic definition was obscure, and it allowed additional time to reinvestigate the source of the ACTH excess with excellent efficacy and acceptable tolerability. Despite KCZ being associated with hepatotoxicity and gastrointestinal complaints, no significant side effect was observed in our patient ( 14 ). Instead of using classic and time-consuming 24h urinary free cortisol concentrations, titration of KCZ was achieved by weekly evaluation of morning salivary cortisol levels, an easy, fast, and reproducible method routinely used in our clinic.

There is no consensus regarding the follow-up of neuroendocrine tumors. ACTH-producing tumors that may relapse or spread after surgery must be strictly followed clinically and biochemically with at least yearly ACTH/cortisol screening and imaging procedures. Some suggest chest and abdomen CTs every six months for the first two years, and then annually. Others recommend somatostatin-receptor-based imaging, such as Ga-68 DOTATE PET/CT ( 15 ).

In summary, we present the challenging case of a young patient with cyclic CS due to an ACTH-producing neuroendocrine tumor of the lung whose clinical manifestations were highly suggestive of EAS, although conflicting test results misled the final diagnosis.
